# Allergic but not autoimmune comorbidities in children with PFAPA: A nationwide matched case–control cohort study

**DOI:** 10.1111/pai.70423

**Published:** 2026-07-14

**Authors:** Yackov Berkun, Eli Magen, Eugene Merzon, Ilan Green, Avivit Golan‐Cohen, Shlomo Vinker, Ariel Israel

**Affiliations:** ^1^ Department of Pediatrics, Hadassah‐Hebrew University Medical Center, Mount Scopus, Faculty of Medicine Hebrew University Jerusalem Israel; ^2^ Leumit Health Services Tel Aviv‐Yafo Israel; ^3^ Medicine A Department, Assuta Ashdod University Hospital, Faculty of Health Sciences Ben‐Gurion University of the Negev Beer‐Sheba Israel; ^4^ Adelson School of Medicine Ariel University Ariel Israel; ^5^ Department of Family Medicine, Gray Faculty of Medical & Health Sciences Tel‐Aviv University Tel Aviv‐Yafo Israel; ^6^ Department of Epidemiology and Preventive Medicine, School of Public Health, Gray Faculty of Medical & Health Sciences Tel Aviv University Ramat Aviv, Tel Aviv Israel

**Keywords:** allergy, asthma, atopy, autoimmune disease, autoinflammatory, PFAPA

## Abstract

**Background:**

Periodic fever, aphthous stomatitis, pharyngitis and cervical adenitis (PFAPA) is the most common autoinflammatory syndrome of childhood. Although immune dysregulation is central to its pathogenesis, the broader burden of allergic and autoimmune comorbidities in PFAPA remains incompletely characterized. We aimed to evaluate the prevalence of allergic and autoimmune diseases in children with PFAPA.

**Methods:**

We conducted a nationwide matched case–control study using electronic health records from Leumit Health Services in Israel between 2000 and 2024. Children with PFAPA were identified using a dedicated ICD‐9 code and matched 1:20 with controls by age, sex, socioeconomic status, ethnicity, and year of first record. Allergic and autoimmune diagnoses were identified using predefined ICD‐9 codes. Odds ratios (ORs) with 95% confidence intervals (CIs) were calculated, with false discovery rate correction for multiple testing.

**Results:**

The cohort included 1641 PFAPA patients and 32,820 matched controls with a mean follow‐up of 8.6 years. PFAPA patients had increased prevalence of asthma (49.6% vs. 37.5%; OR 1.64), allergic rhinitis (15.7% vs. 9.8%; OR 1.71), atopic dermatitis (23.0% vs. 19.9%; OR 1.21), urticaria (18.8% vs. 15.0%; OR 1.32), drug allergy (1.22% vs. 0.57%; OR 2.15), and anaphylaxis (0.55% vs. 0.20%; OR 2.78). In contrast, autoimmune diseases were uncommon and showed no consistent enrichment. Similar associations were observed for diagnoses recorded before PFAPA diagnosis.

**Conclusions:**

PFAPA is associated with a broad atopic comorbidity profile but not with classical autoimmune disease. These findings suggest that PFAPA may represent a distinct immune phenotype in which episodic autoinflammation coexists with atopic susceptibility.

**Key message**

Children with periodic fever syndrome characterized by aphthous stomatitis, pharyngitis, and cervical adenitis (PFAPA) show a consistent enrichment of allergic diseases but not autoimmune conditions. These findings suggest that PFAPA may represent a distinct immune phenotype linking episodic inflammation with allergic susceptibility.

## 1 INTRODUCTION

Periodic fever, aphthous stomatitis, pharyngitis, and cervical adenitis (PFAPA) syndrome is the most common autoinflammatory disorder of childhood and a frequent cause of recurrent fever in pediatric practice.^1^, ^2^ It is characterized by recurrent, self‐limited episodes of high fever accompanied by aphthous stomatitis, pharyngitis, and cervical lymphadenopathy, with complete resolution between attacks.

Despite more than three decades of investigation, the pathogenesis of PFAPA remains incompletely defined. Proposed mechanisms have included infectious triggers, immune dysregulation, and genetic susceptibility, but no unifying etiology has been established.^1^, ^2^ Increasing evidence suggests that PFAPA represents a disorder of innate immune regulation marked by episodic inflammatory activation. Dysregulation within the tonsillar immune microenvironment, potentially triggered by microbial or environmental stimuli, has been demonstrated.^3^ During febrile episodes, excessive production of proinflammatory cytokines, including interleukin‐1 and interleukin‐18, reflects inflammasome activation and Th1‐skewed adaptive immune responses,^4^ aligning PFAPA with other autoinflammatory syndromes.

In hereditary autoinflammatory disorders such as familial Mediterranean fever (FMF), chronic Th1‐dominant inflammation is associated with reduced prevalence of allergic disease, likely through suppression of Th2‐mediated pathways.^5^, ^6^ In contrast, PFAPA involves localized inflammation within tonsillar lymphoid tissue, where persistent immune activation may disrupt mucosal barrier function and immune tolerance, potentially facilitating allergic sensitization.^3^ These observations suggest that PFAPA may exhibit a distinct relationship with atopic disease. However, population‐level data evaluating allergic and autoimmune comorbidities in PFAPA remain limited.

Emerging data indicate that PFAPA is clinically and immunologically heterogeneous, with variable phenotypes, comorbidity patterns, and treatment responses reported in registry studies and single‐center cohorts.^7^, ^8^, ^9^, ^10^ However, existing studies have been limited by small sample sizes, short follow‐up, and heterogeneous methodology, restricting characterization of long‐term comorbidity patterns. Consequently, the prevalence and clinical significance of atopic, allergic, and autoimmune comorbidities in PFAPA remain poorly defined, and it is unclear whether reported associations reflect true disease relationships or surveillance bias.^11^, ^12^, ^13^

To address these gaps, we conducted a nationwide, retrospective matched case–control study using electronic health records from Leumit Health Services in Israel to evaluate comorbidity patterns, medication use, and specialty healthcare utilization among PFAPA patients and matched controls.

## 2 METHODS

### 2.1 Study design

We conducted a nationwide, retrospective matched case–control study using the electronic health record (EHR) database of Leumit Health Services (LHS), a comprehensive healthcare organization providing medical services to approximately 750,000 insured individuals across Israel. The database contains longitudinal demographic, clinical, diagnostic, laboratory, prescription, and healthcare utilization data from community and hospital settings. The study period extended from January 1, 2000, through December 31, 2024.

### 2.2 Healthcare setting

Israel has a universal health insurance system in which all residents are insured by one of four nationwide health maintenance organizations. Insurance is funded through the national social security system using an age‐based allocation formula. Each organization provides care according to a standardized healthcare basket defined and updated annually by the Ministry of Health.

Leumit Health Services (LHS) is one of these organizations and serves a demographically diverse population across Israel through a network of integrated primary care clinics. Its members include individuals from the general population, the Jewish ultra‐orthodox sector, and the Arab population.

Medication dispensing is provided through a network of affiliated pharmacies that fulfill electronic prescriptions from LHS‐affiliated physicians at subsidized cost for LHS members. These pharmacies report all medication purchases, including over‐the‐counter medications, to a central database, ensuring comprehensive capture of dispensing within the organization during active LHS membership. Some over‐the‐counter medication purchases (e.g., certain antihistamines that do not require a prescription) may not be fully captured if made outside the affiliated pharmacy network.

Although insured individuals may switch healthcare organizations at defined intervals, such changes are relatively uncommon due to continuity of care considerations. To reduce bias related to incomplete observation, both cases and controls were required to have continuous active membership from the year of first EHR record through the index date (first PFAPA diagnosis date or assigned date in controls). Cohort follow‐up was censored at death, termination of membership, or the end of data availability.

In a post hoc analysis, we observed no difference in time to censoring between PFAPA patients and controls (3100 ± 1871 vs. 3098 ± 1870 days; *p* = .97; SMD = 0.001), suggesting that differential switching between healthcare organizations is unlikely to have materially influenced the results.

### 2.3 Data extraction and de‐identification

Data were extracted from the LHS central data warehouse using structured query language (SQL) and Python scripts. Patient identifiers, including the national identification number used for patient identification in the electronic health record (EHR), were removed prior to analysis and replaced with study‐specific coded identifiers. All statistical analyses were performed on de‐identified data.

### 2.4 Ethical approval

The study protocol was approved by the Leumit Health Services Institutional Review Board (approval number LEU 05–23). The requirement for informed consent was waived by the Institutional Review Board due to the retrospective design of the study and the use of fully de‐identified data. All data were anonymized prior to analysis in accordance with the approved study protocol and applicable data protection regulations.

### 2.5 Study population

#### 2.5.1 Identification of PFAPA cases

PFAPA cases were identified using the International Classification of Diseases, Ninth Revision (ICD‐9) code designated for PFAPA within the LHS system. This dedicated PFAPA diagnosis code was available throughout the study period. In routine clinical practice, this diagnosis is typically assigned as a chronic condition by pediatricians or pediatric rheumatologists following clinical evaluation and exclusion of major alternative diagnoses, including cyclic neutropenia and monogenic autoinflammatory syndromes, supporting diagnostic specificity.

Only individuals in whom PFAPA was recorded as an active chronic diagnosis and was not subsequently closed or removed from the medical record were included. Within the LHS system, chronic diagnoses remain continuously visible to treating physicians and are routinely reviewed, allowing inaccurate or provisional diagnoses to be discontinued over time.

#### 2.5.2 Selection of matched controls

**FIGURE 1:**
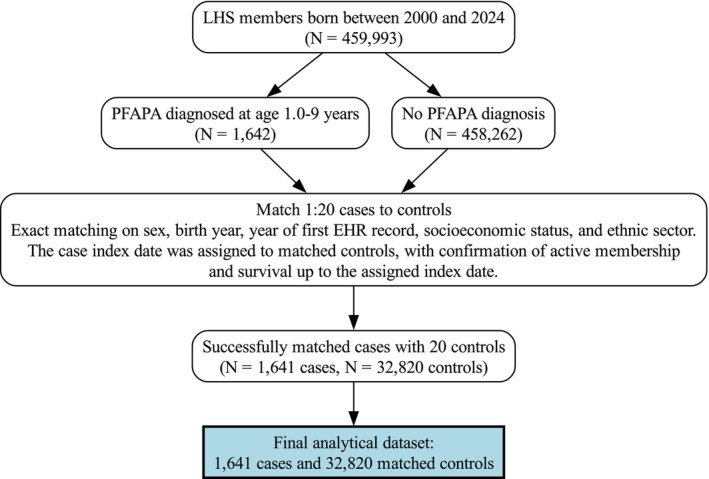
Cohort construction flowchart. Selection of children included in the study from the Leumit Health Services database. Among 459,993 members born between 2000 and 2024, children diagnosed with periodic fever, aphthous stomatitis, pharyngitis, and cervical adenitis were identified and matched 1:20 with controls without the diagnosis using exact matching on sex, birth year, year of first electronic health record entry, socioeconomic status, and ethnic sector. The index date of the case was assigned to matched controls. The final analytical cohort included 1641 cases and 32,820 matched controls.

For each PFAPA case, exactly 20 control individuals without any recorded PFAPA diagnosis were randomly selected from the LHS registry using exact matching on sex, birth year, year of first documented EHR record, socioeconomic status category, and ethnic sector. The date of first recorded PFAPA diagnosis was defined as the index date and assigned to matched controls. Controls were required to have active LHS membership and documented survival through the assigned index date to ensure comparable observation periods. The matching procedure is illustrated in Figure 1.

Of 1642 eligible PFAPA cases, 1641 were successfully matched to 20 controls each, yielding a final analytical cohort of 1641 cases and 32,820 matched controls.

Baseline balance between cases and controls was evaluated using standardized mean differences (SMD), with SMD <0.05 considered indicative of adequate balance, using complete cases, without imputation. All matching variables demonstrated excellent balance (Table 1).

**TABLE 1:** Baseline characteristics of children with PFAPA and matched controls.

*N*	PFAPA (*n* = 1641)	Controls (*n* = 32,820)	OR [95% CI]	SMD
Gender				
Female	637 (38.8%)	12,741 (38.8%)	1.00 [0.90 to 1.11]	
Age (years)	4.198 ± 2.079	4.180 ± 2.081		0.009
Age category				
0–2	387 (23.6%)	7798 (23.8%)	0.99 [0.88 to 1.11]	
3–9	1254 (76.4%)	25,022 (76.2%)	1.01 [0.90 to 1.14]	
BMI (kg/m^2^)	16.82 ± 3.87	16.98 ± 3.88		−0.040
Ethnic sector				
Arab	437 (26.6%)	8722 (26.6%)	1.00 [0.89 to 1.12]	
General	791 (48.2%)	15,839 (48.3%)	1.00 [0.90 to 1.10]	
Jewish Ultra‐orthodox	413 (25.2%)	8259 (25.2%)	1.00 [0.89 to 1.12]	
Socio‐economic status (1–20)	8.355 ± 3.556	8.322 ± 3.550		0.009
Days follow‐up		3131 ± 1871	3129 ± 1870	

*Note*: Baseline demographic and clinical characteristics of PFAPA cases and matched controls at the index date. Continuous variables are presented as mean ± standard deviation and categorical variables as number (percentage). Odds ratios (OR) with 95% confidence intervals (CI) compare PFAPA cases with controls. Standardized mean differences (SMD) were used to assess balance between groups; values close to zero indicate adequate matching. Socioeconomic status is reported on a scale from 1 to 20. Follow‐up time is expressed in days from the index date to the end of observation.

#### 2.5.3 Validation of PFAPA diagnosis accuracy in the cohort

The validity of PFAPA case identification was further supported by an internal validation analysis demonstrating strong enrichment of characteristic pre‐diagnostic clinical patterns (see Table S1). PFAPA patients had markedly higher rates of recurrent tonsillitis/pharyngitis diagnoses compared with matched controls (90.7% vs. 33.1% for ≥1 diagnosis; OR 19.7, 95% CI 16.6–23.5; *p* < .001), with progressively stronger associations at higher frequency thresholds (OR > 30 for ≥5 diagnoses).

Similarly, repeated negative streptococcal throat cultures were substantially more common among PFAPA patients (81.4% vs. 19.0% for ≥1 culture; OR 18.6, 95% CI 16.3–21.2; *p* < .001), again demonstrating a graded increase at higher thresholds (OR > 35 for ≥3 cultures).

In the year following diagnosis, 70% of PFAPA patients received systemic glucocorticoids versus 8% of controls (OR 27.0, 95% CI 24.2–30.4; *p* < .001).

These pronounced and dose‐dependent pre‐diagnostic patterns, together with the expected post‐diagnosis glucocorticoid use, are highly consistent with the typical clinical course and management of PFAPA and support the reliability of PFAPA diagnostic coding within the LHS system.

### 2.6 Follow‐up and outcome assessment

Follow‐up was defined as the interval from the index date to the end of available EHR data, death, or termination of LHS membership, whichever occurred first. Only individuals with documented follow‐up after the index date were included in the analysis.

Chronic comorbidities were considered present if recorded as active and not closed at the time of cohort assembly. Atopic/allergic and autoimmune conditions were identified using predefined ICD‐9 code lists derived from prior literature and clinical relevance. Detailed coding definitions are provided in Tables S1 and S2.

Medication use and healthcare utilization were assessed using pharmacy dispensing and referral records. Medication purchases were evaluated over the entire observation period, including both the pre‐diagnosis and post‐diagnosis intervals. Prescriptions for systemic corticosteroids, antibacterials, antihistamines, leukotriene receptor antagonists, and colchicine were used as proxies for disease management and comorbidity burden.

### 2.7 Statistical analysis

Baseline characteristics were summarized using means with standard deviations for continuous variables and frequencies with percentages for categorical variables. Balance between PFAPA cases and matched controls was assessed using standardized mean differences (SMD), with SMD < 0.05 considered indicative of excellent balance.

Comorbidity analyses were prespecified and categorized into autoimmune and atopic/allergic conditions recorded in the EHR over the entire follow‐up period (Table 2A,B), and separately for diagnoses documented prior to the first recorded PFAPA diagnosis (Table 3A,B). The PFAPA index date was defined as the first documentation of a PFAPA diagnosis. For pre‐index analyses, only diagnoses recorded before this date were included.

**TABLE 2:** Autoimmune and atopic allergic comorbidities at the end of follow‐up.

A. Autoimmune and autoinflammatory conditions					
Condition	PFAPA *N* = 1641	Controls *N* = 32,820	OR [95% CI]	*p* Value	FDR BH
Behcet Disease	2 (0.12%)	2 (0.01%)	20.02 [1.45 to 275.38]	.013	0.078
Henoch‐Schönlein purpura	12 (0.73%)	87 (0.27%)	2.77 [1.38 to 5.10]	.003	0.017
Psoriasis	9 (0.55%)	183 (0.56%)	0.98 [0.44 to 1.91]	1.000	1.000
Hashimoto thyroiditis	5 (0.30%)	107 (0.33%)	0.93 [0.30 to 2.25]	1.000	1.000
Alopecia areata	16 (0.98%)	367 (1.12%)	0.87 [0.49 to 1.44]	.717	1.000
Connective tissue diseases	5 (0.30%)	66 (0.20%)	1.52 [0.48 to 3.73]	.391	0.782
Familial Mediterranean Fever (autoinflammatory)	93 (5.67%)	98 (0.30%)	20.05 [14.86 to 27.06]	<.001	<0.001

B. Atopic/allergic conditions						
Condition	ICD‐9 Codes	PFAPA *N* = 1641	Controls *N* = 32,820	OR [95% CI]	*p* Value	FDR BH
Asthma	493, 493.0–493.92	814 (49.6%)	12,303 (37.5%)	1.64 [1.48 to 1.82]	<.001	<0.001
Allergic rhinitis	477.2, 477.3, 477.4, 477.5, 477.8, 477.80, 477.9	258 (15.7%)	3221 (9.81%)	1.71 [1.49 to 1.97]	<.001	<0.001
Atopic dermatitis	691.80–691.831	378 (23.0%)	6524 (19.9%)	1.21 [1.07 to 1.36]	.002	0.015
Acute urticaria	708.0	309 (18.8%)	4909 (15.0%)	1.32 [1.16 to 1.50]	<.001	<0.001
Food allergies	V15.01‐V15.059	93 (5.67%)	1456 (4.44%)	1.29 [1.03 to 1.61]	.024	0.112
Drug allergies	V14.0‐V14.9	20 (1.22%)	187 (0.57%)	2.15 [1.28 to 3.43]	.003	0.017
Anaphylactic reactions	995.6, 995.60–995.69	9 (0.55%)	65 (0.20%)	2.78 [1.21 to 5.62]	.008	0.045

*Note*: Autoimmune (A) and atopic/allergic (B) comorbidities are shown for PFAPA patients (*N* = 1641) and matched controls (*N* = 32,820) at the end of follow‐up. Values are presented as *n* (%). Odds ratios (ORs) with 95% confidence intervals (CIs) and *p* values were derived from comparisons between matched PFAPA cases and controls. ICD‐9 codes used to define atopic/allergic conditions are listed in panel B.

Abbreviation: FDR, False Discovery Rate, using Benjamini‐Hochberg method.

**TABLE 3:** Autoimmune and atopic allergic comorbidities, before first PFAPA diagnosis.

A. Autoimmune and autoinflammatory conditions					
Condition	PFAPA *N* = 1641	Controls *N* = 32,820	OR [95% CI]	*p* Value	FDR BH
Behcet Disease	3 (0.18%)	36 (0.11%)	1.67 [0.33 to 5.29]	.433	0.965
Henoch‐Schönlein purpura	3 (0.18%)	33 (0.10%)	1.82 [0.36 to 5.81]	.245	0.665
Psoriasis	1 (0.06%)	29 (0.09%)	0.69 [0.02 to 4.16]	1.000	1.000
Hashimoto thyroiditis	4 (0.24%)	126 (0.38%)	0.63 [0.17 to 1.67]	.533	1.000
Alopecia areata	2 (0.12%)	25 (0.08%)	1.60 [0.18 to 6.43]	.371	0.901
Connective tissue diseases	3 (0.18%)	36 (0.11%)	1.67 [0.33 to 5.29]	.433	0.965
Familial Mediterranean Fever (autoinflammatory)	54 (3.29%)	37 (0.11%)	30.14 [19.42 to 47.21]	<.001	<0.001

B. Atopic/allergic conditions						
Condition	ICD‐9 codes	PFAPA *N* = 1641	Controls *N* = 32,820	OR [95% CI]	*p* Value	FDR BH
Asthma	493, 493.0–493.92	663 (40.4%)	9956 (30.3%)	1.56 [1.40 to 1.72]	<.001	<0.001
Allergic rhinitis	477.2, 477.3, 477.4, 477.5, 477.8, 477.80, 477.9	120 (7.31%)	1157 (3.53%)	2.16 [1.76 to 2.63]	<.001	<0.001
Atopic dermatitis	691.80–691.831	280 (17.1%)	4825 (14.7%)	1.19 [1.04 to 1.36]	.009	0.049
Acute urticaria	708.0	186 (11.3%)	3046 (9.28%)	1.25 [1.06 to 1.46]	.006	0.035
Food allergies	V15.01‐V15.059	82 (5.00%)	1248 (3.80%)	1.33 [1.04 to 1.68]	.018	0.089
Drug allergies	V14.0‐V14.9	15 (0.91%)	93 (0.28%)	3.25 [1.74 to 5.65]	<.001	0.001
Anaphylactic reactions	995.6, 995.60–995.69	6 (0.37%)	34 (0.10%)	3.54 [1.21 to 8.54]	.011	0.057

*Note*: Autoimmune (A) and atopic/allergic (B) comorbidities are shown for PFAPA patients (*N* = 1641) and matched controls (*N* = 32,820) before the first PFAPA diagnosis. Values are presented as *n* (%). Odds ratios (ORs) with 95% confidence intervals (CIs) and *p* values were derived from comparisons between matched PFAPA cases and controls. ICD‐9 codes used to define atopic/allergic conditions are listed in panel B.

Abbreviation: FDR, False Discovery Rate, using Benjamini‐Hochberg method.

Associations between PFAPA and each outcome were evaluated by calculating odds ratios (ORs) with 95% confidence intervals (CIs) derived from 2 × 2 contingency tables. Statistical significance was assessed using Fisher's exact test. Because controls were selected using exact matching on sex, birth year, year of first EHR record, socioeconomic status, and ethnicity, and baseline covariates demonstrated excellent balance (all SMD <0.05), these estimates therefore reflect comparisons between well‐balanced matched groups.

Multiple testing within the autoimmune and atopic/allergic comorbidity families was addressed using the Benjamini‐Hochberg false discovery rate procedure, with a two‐sided *q* value <.05 considered statistically significant.

All statistical analyses were performed using R software (version 4.4.0). Two‐sided *p* values <.05 were considered statistically significant unless otherwise specified.

## 3 RESULTS

### 3.1 Study population and baseline characteristics

The cohort construction process is shown in Figure 1. The final analytical cohort included 1641 PFAPA patients and 32,820 matched controls (1:20 matching). Baseline demographic and clinical characteristics were well balanced between groups (Table 1).

The mean age at index date was 4.20 ± 2.08 years in PFAPA patients and 4.18 ± 2.08 years in controls, and 38.8% of participants were female in both groups. Ethnic sector and socioeconomic status were comparable by design. Mean body mass index did not differ significantly between cases and controls (16.82 ± 3.87 vs. 16.98 ± 3.88 kg/m^2^; *p* = .121; SMD = −0.040).

Mean follow‐up duration was similar between groups (3131 ± 1871 days vs. 3129 ± 1870 days), corresponding to approximately 8.6 years of observation.

### 3.2 Autoimmune and autoinflammatory comorbidities

Familial Mediterranean fever (FMF), an autoinflammatory condition with known clinical overlap with PFAPA, was more prevalent among PFAPA patients than controls both prior to the index date and during overall follow‐up (Tables 2A and 3A).

Apart from FMF, autoimmune diseases were uncommon in both groups and did not demonstrate consistent enrichment among PFAPA patients (Table 2A). Although Henoch‐Schönlein purpura remained statistically significant after correction, the absolute number of cases was small and no consistent pattern of autoimmune enrichment was observed. Other autoimmune conditions, including psoriasis, Hashimoto thyroiditis, alopecia areata, and connective tissue diseases, occurred at similar frequencies in PFAPA patients and controls.

A similar absence of enrichment for autoimmune conditions was observed in the pre‐diagnosis period (Table 3A), with no autoimmune outcome demonstrating consistent or statistically significant differences after correction for multiple testing.

### 3.3 Atopic and allergic comorbidities

PFAPA patients demonstrated a consistent and broad increase in atopic and allergic comorbidities compared with matched controls (Table 2B). Respiratory allergic diseases were significantly more prevalent, including asthma (49.6% vs. 37.5%; OR 1.64, 95% CI 1.48–1.82; *p* < .001) and allergic rhinitis (15.7% vs. 9.81%; OR 1.71, 95% CI 1.49–1.97; *p* < .001).

Dermatologic allergic conditions were also more common among PFAPA patients, including atopic dermatitis (23.0% vs. 19.9%; OR 1.21, 95% CI 1.07–1.36; *p* = .002) and acute urticaria (18.8% vs. 15.0%; OR 1.32, 95% CI 1.16–1.50; *p* < .001). PFAPA patients also had higher prevalence of food allergy (5.67% vs. 4.44%; OR 1.29, 95% CI 1.03–1.61; *p* = .024), drug allergy (1.22% vs. 0.57%; OR 2.15, 95% CI 1.28–3.43; *p* = .003), and anaphylactic reactions (0.55% vs. 0.20%; OR 2.78, 95% CI 1.21–5.62; *p* = .008). After Benjamini–Hochberg false discovery rate correction, asthma, allergic rhinitis, atopic dermatitis, acute urticaria, drug allergy, and anaphylaxis remained statistically significant, whereas food allergy did not.

To evaluate the possibility of global surveillance bias, we examined several non‐atopic diagnoses as negative control outcomes (Table S2). ADHD, autism spectrum disorder, and myopia did not differ significantly between PFAPA patients and controls. Celiac disease showed a nominal difference toward lower prevalence in PFAPA patients that did not remain significant after false discovery rate correction. These findings do not support a generalized increase in diagnostic labeling among PFAPA patients.

Pre‐diagnosis analyses yielded similar findings (Table 3B). Asthma (OR 1.56), allergic rhinitis (OR 2.16), atopic dermatitis (OR 1.19), acute urticaria (OR 1.25), and drug allergy (OR 3.25) were significantly more prevalent among children with PFAPA even before the first recorded PFAPA diagnosis, with most associations remaining significant after FDR correction.

The presence of multiple atopic diagnoses prior to the first recorded PFAPA diagnosis suggests that the observed associations are unlikely to be attributable solely to post‐diagnosis surveillance or differential healthcare utilization.

### 3.4 Medication use

Medication utilization differed markedly between groups (Table 4A). Systemic corticosteroid use was substantially higher among PFAPA patients (90.0% vs. 41.8%; OR 12.5, 95% CI 10.7–14.8; *p* < .001). Colchicine use was also more frequent in PFAPA patients (8.2% vs. 0.31%; OR 28.2, 95% CI 21.7–36.7; *p* < .001).

**TABLE 4:** Medication use and specialty healthcare utilization among PFAPA patients and matched controls.

	PFAPA N = 1641	Controls N = 32,820	OR [95% CI]	*p* Value	FDR BH
Medications					
Corticosteroids (H02)	1477 (90.0%)	13,719 (41.8%)	12.5 [10.7 to 14.8]	<.001	<0.001
Colchicine (M04AC01)	134 (8.2%)	103 (0.31%)	28.2 [21.7 to 36.7]	<.001	<0.001
Antibacterials (J01)	1620 (98.7%)	30,476 (92.9%)	5.93 [3.85 to 9.14]	<.001	<0.001
Antihistamines (R06)	1393 (84.9%)	24,768 (75.5%)	1.83 [1.59 to 2.10]	<.001	<0.001
Leukotriene Receptor Antagonists (R03DC)	186 (11.3%)	2211 (6.7%)	1.77 [1.51 to 2.07]	<.001	<0.001
Sympathomimetics (R01BA)	811 (49.4%)	12,659 (38.6%)	1.56 [1.41 to 1.72]	<.001	<0.001
Anticholinergics (R03BB)	302 (18.4%)	3679 (11.2%)	1.79 [1.57 to 2.03]	<.001	<0.001
Specialty visits					
Otorhinolaryngology	1162 (70.8%)	17,581 (53.6%)	2.10 [1.89 to 2.34]	<.001	<0.001
Rheumatology	65 (4.0%)	149 (0.45%)	9.04 [6.73 to 12.2]	<.001	<0.001
Pediatric Rheumatology	25 (1.5%)	31 (0.09%)	16.4 [9.64 to 27.8]	<.001	<0.001
Pediatric Gastroenterology	212 (12.9%)	2319 (7.1%)	1.95 [1.68 to 2.27]	<.001	<0.001
Dermatology	976 (59.5%)	16,714 (50.9%)	1.41 [1.28 to 1.56]	<.001	<0.001
Allergy and Clinical Immunology	194 (11.8%)	2638 (8.04%)	1.53 [1.31 to 1.79]	<.001	<0.001
Pulmonology	55 (3.35%)	568 (1.73%)	1.97 [1.49 to 2.61]	<.001	<0.001

*Note*: Medication dispensing and specialty consultations during follow‐up among children with PFAPA and matched controls. Values are presented as number (percentage) of patients with at least one recorded purchase of a medication within each class during the observation period, including both pre‐index and post‐index intervals. Odds ratios (OR) with 95% confidence intervals (CI) compare PFAPA patients with controls. Medication classes are defined using Anatomical Therapeutic Chemical (ATC) codes. *p* values were calculated using Fisher's exact test, and false discovery rate (FDR) correction was applied using the Benjamini‐Hochberg procedure.

PFAPA patients had increased use of antibacterials (98.7% vs. 92.9%; OR 5.93, 95% CI 3.85–9.14; *p* < .001), antihistamines (84.9% vs. 75.5%; OR 1.83, 95% CI 1.59–2.10; *p* < .001), and leukotriene receptor antagonists (11.3% vs. 6.7%; OR 1.77, 95% CI 1.51–2.07; *p* < .001).

Additional increases were observed for sympathomimetics and anticholinergic respiratory medications. All medication comparisons remained significant after false discovery rate correction.

### 3.5 Specialty healthcare utilization

Specialty consultation rates were consistently higher among PFAPA patients (Table 4B). PFAPA patients were more likely to be evaluated by otorhinolaryngology (70.8% vs. 53.6%; OR 2.10, 95% CI 1.89–2.34; *p* < .001), dermatology (59.5% vs. 50.9%; OR 1.41, 95% CI 1.28–1.56; *p* < .001), allergy and clinical immunology (11.8% vs. 8.04%; OR 1.53, 95% CI 1.31–1.79; *p* < .001), and pulmonology (3.35% vs. 1.73%; OR 1.97, 95% CI 1.49–2.61; *p* < .001).

Marked differences were also observed for rheumatology and pediatric rheumatology consultations (OR 9.04 and OR 16.4, respectively; both *p* < .001). Gastroenterology visits were more frequent in PFAPA patients (12.9% vs. 7.1%; OR 1.95, 95% CI 1.68–2.27; *p* < .001).

All specialty utilization comparisons remained statistically significant after correction for multiple testing.

## 4 DISCUSSION

In this nationwide, healthcare‐based matched case–control study, PFAPA was associated with a broad and consistent increase in atopic and allergic comorbidities across multiple organ systems, whereas classical autoimmune diseases were uncommon. These findings suggest that PFAPA may reflect shared immunologic susceptibility or immune regulatory pathways characterized by the coexistence of episodic autoinflammation and heightened susceptibility to allergic disease rather than progression toward chronic autoimmunity. These observations describe epidemiologic associations and should not be interpreted as evidence of causality or direct mechanistic linkage.

A central finding of this study is the substantially increased prevalence of allergic disorders among PFAPA patients, including asthma, allergic rhinitis, atopic dermatitis, urticaria, food allergy, drug allergy, and anaphylaxis. The parallel increase in allergy‐related medication use indicates that these conditions are clinically significant and require active management. The consistency of associations across diverse allergic phenotypes supports a generalized atopic predisposition rather than isolated comorbidities and extends prior evidence suggesting complex immune dysregulation in PFAPA involving both innate and adaptive pathways.^1^, ^14^, ^15^

Previous studies evaluating comorbidity patterns in PFAPA have yielded heterogeneous results. The Swedish cohort described by Rydenman et al.^11^ did not systematically assess allergic outcomes, whereas the Finnish case–control study by Lantto et al.^13^ and the long‐term follow‐up cohort reported by Wurster et al.^12^ did not identify increased allergic disease prevalence. These discrepancies may reflect differences in sample size, follow‐up duration, disease definitions, and geographic or environmental factors. Improved diagnostic awareness and evolving allergy practices may also contribute to greater recognition of allergic disease in contemporary cohorts. The large scale, extended follow‐up, and combined use of diagnostic and medication‐based proxies in the present study provide enhanced statistical power and internal validity.

The enrichment of allergic disease in PFAPA contrasts with patterns observed in Familial Mediterranean Fever (FMF), in which allergic conditions are relatively uncommon. In the present cohort, FMF itself was more prevalent among PFAPA patients, consistent with known clinical overlap between these conditions.^5^, ^6^ In FMF, persistent Th1‐dominant inflammation and chronic cytokine activation are thought to suppress Th2‐mediated immune responses. Our findings do not support a strictly antagonistic Th1–Th2 framework and instead suggest that autoinflammatory and atopic processes may coexist within the same individuals.^14^ In PFAPA, episodic innate immune activation does not appear to preclude type 2 immune responses. Recurrent tonsillar inflammation could theoretically influence mucosal immune regulation; however, dedicated mechanistic studies are required to clarify whether such pathways contribute to the observed associations.^2^, ^3^

Accumulating molecular and genetic data suggest that PFAPA may represent a clinically distinct immunologic phenotype characterized by episodic IL‐1–driven inflammation with Th1 activation and resolution between attacks.^15^ Shared susceptibility loci linking PFAPA with Behçet's disease and recurrent aphthous stomatitis further implicate pathways regulating innate immunity and mucosal inflammation.^16^ Within this context, allergic comorbidity may reflect overlapping susceptibility rather than a distinct causal pathway.

Autoimmune diseases were rare in our cohort and did not demonstrate consistent enrichment, consistent with prior cohort studies^13^, ^17^ and original diagnostic criteria emphasizing exclusion of autoimmunity.^18^ Although isolated case reports have described autoimmune manifestations such as autoimmune hepatitis type 2,^19^ these events remain uncommon and are most likely coincidental. This pattern contrasts with FMF, in which 12%–21% of patients develop additional inflammatory conditions,^20^, ^21^ including vasculitis and inflammatory bowel disease in pediatric populations.^22^ FMF is characterized by persistent subclinical inflammation between attacks,^23^ which may impair immune tolerance and promote autoimmunity.^24^ In PFAPA, normalization of inflammatory markers between episodes suggests absence of sustained immune activation,^13^ providing a plausible explanation for the low burden of autoimmune disease.^12^

PFAPA patients demonstrated substantially higher healthcare utilization, including increased use of antibiotics, corticosteroids, and specialty services. These estimates reflect exposure at least once during follow‐up. Elevated antibacterial use likely reflects empirical treatment of febrile episodes before recognition of the syndrome,^25^ whereas increased use of allergy‐related medications corresponds to the observed atopic burden.^26^ These findings underscore the diagnostic challenges associated with PFAPA and highlight the importance of early recognition to reduce unnecessary antibiotic exposure and fragmented care.

Clinicians should maintain vigilance for asthma, allergic rhinitis, food allergy, and atopic dermatitis in children with PFAPA. Early identification may improve symptom control and quality of life. Management strategies should consider both inflammatory and allergic components, and multidisciplinary care may be beneficial for patients with complex presentations.

High antibiotic exposure was observed in PFAPA patients. Although antibiotic exposure has been linked to allergic outcomes in other settings^27^, ^28^, ^29^, ^30^, ^31^, ^32^ the presence of atopic diagnoses prior to PFAPA recognition suggests that antibiotic exposure alone is unlikely to account for the observed associations.

This study has several strengths, including its nationwide design, large matched control cohort, stringent case definition relying on a dedicated diagnosis code, long follow‐up, and comprehensive assessment of comorbidities and medication use. Limitations include reliance on administrative coding, potential residual misclassification, and the lack of detailed disease severity and immunologic data.

An important concern in observational studies is detection bias arising from increased healthcare utilization. In our cohort, PFAPA patients demonstrated greater healthcare contact. However, enrichment of several atopic diagnoses was already evident prior to the first documented PFAPA diagnosis, and multiple non‐atopic control conditions—including ADHD, autism spectrum disorder, psoriasis, and myopia—were not enriched among PFAPA patients. These findings argue against a generalized diagnostic inflation effect and suggest that the observed associations are unlikely to be explained solely by post‐diagnosis surveillance. Although residual detection bias cannot be entirely excluded, the consistency of the pre‐index findings and the selective enrichment of allergic, but not unrelated, conditions support the robustness of the observed associations.

## 5 CONCLUSION

This large nationwide healthcare‐based study demonstrates that PFAPA is associated with a broad atopic and allergic comorbidity profile, whereas classical autoimmune diseases remain uncommon. These findings suggest that PFAPA may represent a distinct immune phenotype in which episodic autoinflammation coexists with atopic susceptibility. Recognition of this dual pattern has implications for clinical evaluation and management. Further prospective and mechanistic studies are needed to clarify the pathways linking mucosal immunity, autoinflammation, and allergic disease in this common pediatric syndrome.

## AUTHOR CONTRIBUTIONS

**Eugene Merzon:** Conceptualization; investigation; writing – review and editing. **Ilan Green:** Writing – review and editing. **Shlomo Vinker:** Writing – review and editing. **Eli Magen:** Conceptualization; investigation; writing – original draft. **Ariel Israel:** Writing – original draft; writing – review and editing; conceptualization; investigation; formal analysis; methodology. **Avivit Golan‐Cohen:** Writing – review and editing. **Yackov Berkun:** Conceptualization; investigation; writing – original draft.

## CONFLICT OF INTEREST STATEMENT

None.

## Supporting information


Table S1.

## Data Availability

The data that support the findings of this study are not publicly available due to Israel Ministry of Health regulations governing patient privacy and institutional data protection policies. De‐identified data may be made available from the corresponding author upon reasonable request, subject to approval by the Leumit Health Services Institutional Review Board and in accordance with applicable data protection regulations.
