# Supplementary material for: Allergic but not autoimmune comorbidities in children with PFAPA: A nationwide matched case–control cohort study
Source: Pediatr Allergy Immunol. 2026 Jul 14;37(7):e70423. doi: 10.1111/pai.70423 (PMC13370066; doi:10.1111/pai.70423)
Supplement: Supplementary file 1 — Table S1. [file PAI-37-e70423-s001.docx]

**Supplementary Material for**

**Allergic but not autoimmune comorbidities in children with PFAPA: a nationwide matched cohort study**

Yackov Berkun M.D.^1,2^, Eli Magen M.D.^1,3^, Eugene Merzon M.D.^1,4^, Ilan Green M.D.^1,5^, Avivit Golan-Cohen M.D.^1,5^, Shlomo Vinker M.D.^1,5^, Ariel Israel M.D., Ph.D.^1,6^

Contents

[Supplementary Table S1. Validation of PFAPA diagnostic coding using clinical patterns before diagnosis and medication use after diagnosis. 2](#_Toc223960338)

[Supplementary Table S2. Negative control diagnoses used to evaluate potential surveillance bias. 3](#_Toc223960339)

## Supplementary Table S1. Validation of PFAPA diagnostic coding using pre-diagnostic clinical patterns across ethnic sectors.

|  | **PFAPA n (%)** | **Controls n (%)** | **Odds ratio (95% CI)** | **P value** |
| --- | --- | --- | --- | --- |
| **All individuals** | **n=1641** | **n=32820** |  |  |
| **Tonsillitis/pharyngitis diagnoses ≥ 1** | 1,488 (90.7 %) | 10,850 (33.1 %) | 19.69 [16.64–23.45] | 0.0E+00 |
| **Tonsillitis/pharyngitis diagnoses ≥ 2** | 1,280 (78.0 %) | 5,057 (15.4 %) | 19.47 [17.24–22.01] | 0.0E+00 |
| **Tonsillitis/pharyngitis diagnoses ≥ 3** | 1,070 (65.2 %) | 2,472 (7.53 %) | 22.99 [20.59–25.72] | 0.0E+00 |
| **Tonsillitis/pharyngitis diagnoses ≥ 4** | 878 (53.5 %) | 1,285 (3.92 %) | 28.25 [25.19–31.66] | 0.0E+00 |
| **Tonsillitis/pharyngitis diagnoses ≥ 5** | 672 (41.0 %) | 690 (2.10 %) | 32.26 [28.46–36.65] | 0.0E+00 |
| **Negative streptococcal throat cultures ≥ 1** | 1,335 (81.4 %) | 6,241 (19.0 %) | 18.57 [16.34–21.17] | 0.0E+00 |
| **Negative streptococcal throat cultures ≥ 2** | 978 (59.6 %) | 1,853 (5.65 %) | 24.65 [22.06–27.52] | 0.0E+00 |
| **Negative streptococcal throat cultures ≥ 3** | 707 (43.1 %) | 666 (2.03 %) | 36.53 [32.18–41.49] | 0.0E+00 |
| **Negative streptococcal throat cultures ≥ 4** | 460 (28.0 %) | 285 (0.87 %) | 44.43 [37.81–52.28] | 0.0E+00 |
| **Negative streptococcal throat cultures ≥ 5** | 294 (17.9 %) | 119 (0.36 %) | 59.98 [47.99–75.21] | 8.5E-297 |
| **Jewish General** | **n=791** | **n=15,839** |  |  |
| **Tonsillitis/pharyngitis diagnoses ≥ 1** | 718 (90.8 %) | 5,356 (33.8 %) | 19.25 [15.07–24.89] | 9.6E-236 |
| **Tonsillitis/pharyngitis diagnoses ≥ 2** | 633 (80.0 %) | 2,572 (16.2 %) | 20.65 [17.24–24.89] | 0.0E+00 |
| **Tonsillitis/pharyngitis diagnoses ≥ 3** | 536 (67.8 %) | 1,294 (8.17 %) | 23.62 [20.09–27.82] | 0.0E+00 |
| **Tonsillitis/pharyngitis diagnoses ≥ 4** | 443 (56.0 %) | 671 (4.24 %) | 28.76 [24.43–33.87] | 0.0E+00 |
| **Tonsillitis/pharyngitis diagnoses ≥ 5** | 354 (44.8 %) | 365 (2.30 %) | 34.31 [28.74–41.00] | 8.5E-298 |
| **Negative streptococcal throat cultures ≥ 1** | 680 (86.0 %) | 3,599 (22.7 %) | 20.82 [16.96–25.78] | 1.0E-294 |
| **Negative streptococcal throat cultures ≥ 2** | 522 (66.0 %) | 1,118 (7.06 %) | 25.52 [21.74–30.07] | 0.0E+00 |
| **Negative streptococcal throat cultures ≥ 3** | 397 (50.2 %) | 419 (2.65 %) | 37.05 [31.15–44.15] | 0.0E+00 |
| **Negative streptococcal throat cultures ≥ 4** | 258 (32.6 %) | 182 (1.15 %) | 41.59 [33.59–51.64] | 3.8E-236 |
| **Negative streptococcal throat cultures ≥ 5** | 167 (21.1 %) | 85 (0.54 %) | 49.49 [37.47–66.05] | 3.3E-162 |
| **Jewish Ultra-Orthodox** | **n=413** | **n=8,259** |  |  |
| **Tonsillitis/pharyngitis diagnoses ≥ 1** | 371 (89.8 %) | 2,322 (28.1 %) | 22.58 [16.31–31.96] | 1.8E-145 |
| **Tonsillitis/pharyngitis diagnoses ≥ 2** | 314 (76.0 %) | 1,109 (13.4 %) | 20.44 [16.10–26.10] | 1.2E-169 |
| **Tonsillitis/pharyngitis diagnoses ≥ 3** | 259 (62.7 %) | 541 (6.55 %) | 23.97 [19.19–30.01] | 5.4E-175 |
| **Tonsillitis/pharyngitis diagnoses ≥ 4** | 218 (52.8 %) | 290 (3.51 %) | 30.67 [24.32–38.73] | 1.9E-172 |
| **Tonsillitis/pharyngitis diagnoses ≥ 5** | 163 (39.5 %) | 160 (1.94 %) | 32.94 [25.42–42.74] | 5.9E-138 |
| **Negative streptococcal throat cultures ≥ 1** | 358 (86.7 %) | 1,741 (21.1 %) | 24.34 [18.19–33.11] | 1.4E-168 |
| **Negative streptococcal throat cultures ≥ 2** | 271 (65.6 %) | 528 (6.39 %) | 27.91 [22.28–35.14] | 4.2E-192 |
| **Negative streptococcal throat cultures ≥ 3** | 202 (48.9 %) | 186 (2.25 %) | 41.46 [32.35–53.28] | 1.3E-179 |
| **Negative streptococcal throat cultures ≥ 4** | 129 (31.2 %) | 77 (0.93 %) | 48.15 [35.15–66.36] | 7.3E-124 |
| **Negative streptococcal throat cultures ≥ 5** | 82 (19.9 %) | 26 (0.31 %) | 78.10 [49.07–128.97] | 2.5E-88 |
| **Arab** | **n=437** | **n=8,722** |  |  |
| **Tonsillitis/pharyngitis diagnoses ≥ 1** | 399 (91.3 %) | 3,172 (36.4 %) | 18.37 [13.11–26.41] | 1.1E-122 |
| **Tonsillitis/pharyngitis diagnoses ≥ 2** | 333 (76.2 %) | 1,376 (15.8 %) | 17.09 [13.56–21.66] | 8.3E-161 |
| **Tonsillitis/pharyngitis diagnoses ≥ 3** | 275 (62.9 %) | 637 (7.30 %) | 21.53 [17.36–26.74] | 4.9E-176 |
| **Tonsillitis/pharyngitis diagnoses ≥ 4** | 217 (49.7 %) | 324 (3.71 %) | 25.54 [20.43–31.94] | 1.2E-161 |
| **Tonsillitis/pharyngitis diagnoses ≥ 5** | 155 (35.5 %) | 165 (1.89 %) | 28.46 [22.01–36.87] | 6.4E-126 |
| **Negative streptococcal throat cultures ≥ 1** | 297 (68.0 %) | 901 (10.3 %) | 18.40 [14.82–22.93] | 2.4E-167 |
| **Negative streptococcal throat cultures ≥ 2** | 185 (42.3 %) | 207 (2.37 %) | 30.15 [23.69–38.41] | 1.4E-150 |
| **Negative streptococcal throat cultures ≥ 3** | 108 (24.7 %) | 61 (0.70 %) | 46.53 [33.00–66.02] | 1.3E-103 |
| **Negative streptococcal throat cultures ≥ 4** | 73 (16.7 %) | 26 (0.30 %) | 66.96 [41.70–110.31] | 1.4E-76 |
| **Negative streptococcal throat cultures ≥ 5** | 45 (10.3 %) | 8 (0.09 %) | 124.89 [57.77–306.4] | 2.3E-52 |

Clinical patterns recorded during the year preceding the first documented PFAPA diagnosis were compared between PFAPA patients and matched controls. Frequencies of tonsillitis/pharyngitis diagnoses and negative streptococcal throat cultures were evaluated using increasing frequency thresholds (≥1 to ≥5 events). Results are shown for the overall cohort and stratified by ethnic sector. Values are presented as n (%). Odds ratios (OR) and 95% confidence intervals (CI) were calculated from contingency comparisons between PFAPA patients and matched controls.

## Supplementary Table S2. Negative control diagnoses used to evaluate potential surveillance bias.

|  | **case** | **control** | **OR (95% CI)** | **p value** | **FDR BH** |
| --- | --- | --- | --- | --- | --- |
| **N** | 1,641 | 32,820 |  |  |  |
| **ADHD** | 287 (17.5 %) | 5,640 (17.2 %) | 1.02 [0.89 to 1.16] | 0.738 | 1.000 |
| **Autism spectrum disorder** | 32 (1.95 %) | 657 (2.00 %) | 0.97 [0.66 to 1.39] | 1.000 | 1.000 |
| **Celiac Disease** | 5 (0.30 %) | 256 (0.78 %) | 0.39 [0.12 to 0.92] | 0.027 | 0.124 |
| **Psoriasis** | 9 (0.55 %) | 183 (0.56 %) | 0.98 [0.44 to 1.91] | 1.000 | 1.000 |
| **Myopia** | 201 (12.2 %) | 3,562 (10.9 %) | 1.15 [0.98 to 1.34] | 0.081 | 0.281 |
| **Contact dermatitis** | 431 (26.3 %) | 8,131 (24.8 %) | 1.08 [0.96 to 1.21] | 0.178 | 0.460 |

Selected non-atopic diagnoses unlikely to be biologically related to PFAPA were analyzed as negative control outcomes. Prevalence of these diagnoses was compared between PFAPA patients and matched controls. Values are presented as n (%). Odds ratios (ORs) with 95% confidence intervals (CIs) were calculated from comparisons between PFAPA patients and controls. P values were adjusted for multiple comparisons using the Benjamini-Hochberg false discovery rate (FDR).
